# Conditional Tabular Generative Adversarial Based Intrusion Detection System for Detecting Ddos and Dos Attacks on the Internet of Things Networks

**DOI:** 10.3390/s23125644

**Published:** 2023-06-16

**Authors:** Basim Ahmad Alabsi, Mohammed Anbar, Shaza Dawood Ahmed Rihan

**Affiliations:** 1 Applied College, Najran University, Kind Abdulaziz Street, Najran P.O. Box 1988, Saudi Arabiasdrihan@nu.edu.sa (S.D.A.R.); 2 National Advanced IPv6 (NAv6) Centre, Universiti Sains Malaysia, Gelugor 11800, Penang, Malaysia

**Keywords:** internet of things, distributed denial of service, denial of service, intrusion detection system, conditional tabular generative adversarial network

## Abstract

The increasing use of Internet of Things (IoT) devices has led to a rise in Distributed Denial of Service (DDoS) and Denial of Service (DoS) attacks on these networks. These attacks can have severe consequences, resulting in the unavailability of critical services and financial losses. In this paper, we propose an Intrusion Detection System (IDS) based on a Conditional Tabular Generative Adversarial Network (CTGAN) for detecting DDoS and DoS attacks on IoT networks. Our CGAN-based IDS utilizes a generator network to produce synthetic traffic that mimics legitimate traffic patterns, while the discriminator network learns to differentiate between legitimate and malicious traffic. The syntactic tabular data generated by CTGAN is employed to train multiple shallow machine-learning and deep-learning classifiers, enhancing their detection model performance. The proposed approach is evaluated using the Bot-IoT dataset, measuring detection accuracy, precision, recall, and F1 measure. Our experimental results demonstrate the accurate detection of DDoS and DoS attacks on IoT networks using the proposed approach. Furthermore, the results highlight the significant contribution of CTGAN in improving the performance of detection models in machine learning and deep learning classifiers.

## 1. Introduction

The Internet of Things (IoT) has become more widespread in recent years, with applications ranging from smart homes and wearable technology to factory automation. However, due to their increased use, IoT devices are increasingly vulnerable to Denial-of-Service (DoS) and Distributed Denial-of-Service (DDoS) attacks and other novel cyber threats. DDoS/DoS attacks on IoT networks aim to make the services and resources of the targeted network or devices inaccessible to legitimate users. This is accomplished by inundating the network or devices with an enormous volume of malicious traffic, depleting their available resources such as bandwidth, processing power, or memory. Traditional intrusion detection systems (IDS) have difficulty retaining and identifying these threats because of the amount and diversity of IoT data [1].

With more and more devices connecting to networks and inadequate protections in place, DDoS attacks on IoT infrastructure have become more common and destructive. The purpose of these kinds of attacks is to employ many infected machines to flood a network or server with traffic, rendering it unavailable to its legitimate users. IoT networks are vulnerable to DDoS attacks because of their limited processing capacity, lack of security measures, and the possibility of broad infiltration, which might jeopardize critical data and cause disruptions in services like healthcare and transportation. Developing IDS systems that can identify DDoS attacks is vital for ensuring the availability and security of IoT networks [2].

According to a report by Kaspersky [3], the number of DDoS attacks targeting IoT devices increased by 9.5 times between 2017 and 2018. In 2019, IoT devices were found to be involved in 32.7% of all DDoS attacks worldwide.

Another report by NETSCOUT [4] revealed that in the first half of 2021, IoT devices were involved in 29% of all DDoS attacks globally. The report also highlighted that there was a significant increase in the number of amplification attacks that used IoT devices, which rose by 1630% compared to the same period in 2020.

These statistics indicate that IoT devices are becoming an increasingly popular target for DDoS and DoS attacks, and organizations must take proactive measures to protect their networks and devices from these attacks.

To detect DDoS/DoS attacks in IoT networks, traditional IDS utilize approaches such as statistical anomaly detection, signature-based detection, and machine learning-based detection. However, the detection of DDoS/DoS attacks in IoT networks poses a significant challenge for traditional intrusion detection systems (IDS). These systems typically employ techniques such as statistical anomaly detection, signature-based detection, and machine learning-based detection. However, the unique characteristics of IoT networks, including a vast number of interconnected devices, varied communication protocols, and heterogeneous traffic patterns, contribute to the complexity of detecting malicious activities. Traditional IDS methods, which were primarily designed for conventional networks, struggle to cope with the dynamic and unpredictable nature of IoT environments [5].

This study highlights the critical need to investigate new ways to improve IDS detection capabilities for IoT networks. Researchers can make strides in developing more reliable and accurate techniques for identifying and mitigating DDoS/DoS assaults by addressing the challenges posed by the enormous quantity and variety of IoT traffic. Safeguarding IoT networks and devices through enhanced detection methods is essential for maintaining the trustworthiness and security of many IoT applications and services.

To aid in the development of efficient DDoS/DoS detection approaches for IoT networks, researchers can push the limits of existing research, explore new detection algorithms, utilize advanced machine learning algorithms such as CTGAN, or adapt existing methods to suit the distinctive features of IoT traffic. Ultimately, these advancements will strengthen the security and resilience of IoT systems, making them more resistant to and better equipped to handle the increasing dangers of DDoS/DoS attacks.

The key contributions of this research work can be summarized as follows:

- An approach that leverages CTGAN for accurate identification of DDoS and DoS attacks in IoT networks. The proposed approach utilizes the power of generative adversarial networks to synthesize realistic network traffic data, enabling more effective detection and classification of malicious activities.
- Conducting an extensive evaluation of the classification performance of various shallow machine learning (ML) and deep learning (DL) models. By leveraging the synthetic dataset generated by CTGAN, this research pioneers a comprehensive assessment of different ML and DL algorithms, providing insights into their strengths and weaknesses in detecting DDoS/DoS attacks in IoT networks. This evaluation contributes to the understanding of the most effective models for accurate attack classification.Furthermore, this evaluation serves as a valuable resource for future researchers in the same field, aiding them in identifying the optimal combination of machine ML or DL techniques in conjunction with CTGAN.
- Addressing the issue of extreme class imbalance in the Bot-IoT dataset through the utilization of synthetic data generation. The research proposes the use of CTGAN to generate synthetic data that represents the minority class of DDoS and DoS attacks. By augmenting the dataset with synthetic samples, this approach helps alleviate the challenges associated with imbalanced training data, enhancing the performance and robustness of detection models.

By presenting these contributions, the research contributes to the development of more effective and reliable intrusion detection systems tailored to the unique characteristics of IoT environments.

The remaining sections of the paper are organized as follows: Section 2 introduces the background of this research. Section 3 discusses related works. Section 4 outlines the proposed approach. Section 5 showcases the experimental results. Finally, Section 6 outlines the conclusions and future works.

## 2. Background

This section provides an overview of DDoS and DoS attacks on IoT networks and briefly explains the Conditional Tabular Generative Adversarial Network (CTGAN).

### 2.1. Distributed Denial of Service (DDoS) and Denial of Service (DoS)

IoT networks are highly susceptible to cyber-attacks, with DDoS and DoS attacks being two of the most prevalent types. These attacks can cause significant disruptions to critical services, resulting in financial loss and damage to the reputation of affected organizations. The vulnerability of IoT devices is a significant contributing factor to these attacks, as they often lack security measures and computing resources. DDoS attacks involve a coordinated effort by multiple devices to flood a network or server with traffic, rendering it inaccessible to legitimate users. This is often accomplished by utilizing compromised devices, such as those infected with malware or bots. In contrast, DoS attacks involve a single device or a small group of devices overwhelming the network with traffic, causing it to become unavailable. The key differentiator between DDoS and DoS attacks is the number of devices employed to carry out the attack [6].

There are several ways in which DDoS and DoS attacks against IoT devices are distinct from DDoS and DoS attacks on conventional networks. One key distinction between conventional computing devices and IoT devices is the latter’s generally lower processing power and memory. This makes them more susceptible to resource depletion attacks, in which the target is subjected to such a high volume of requests or traffic that it becomes incapacitated. In addition, it might be more difficult to identify and counteract attacks in real-time when dealing with IoT devices since they may be dispersed across a large geographical region and linked through a variety of network protocols and communication channels. The necessity for strong security measures to defend against DDoS and DoS attacks will only increase as IoT devices continue to spread and become more ingrained in essential infrastructure and day-to-day life [7,8].

Furthermore, the low-cost and easy-to-use nature of IoT devices, coupled with a lack of emphasis on security during their design, makes them an attractive target for cybercriminals to exploit their vulnerabilities. Many IoT devices have default login credentials that are either easily guessable or readily accessible, enabling attackers to gain unauthorized entry to these devices and exploit them for malicious activities, including the initiation of DDoS and DoS attacks.

Generally, DDoS/DoS attacks in IoT networks pose unique challenges, with certain types of attacks being more prevalent. One example is IoT Botnet-based DDoS attacks [9]. These attacks exploit the large number of interconnected IoT devices to launch massive-scale attacks. The prevalence of these attacks is due to the vulnerability of IoT devices, their widespread deployment, and the resource constraints of IoT devices. Detecting and mitigating such attacks in real-time is challenging due to limited device capabilities and the heterogeneity of IoT devices and communication protocols. Table 1 shows attacks on IoT networks other than DDoS and DoS [10].

### 2.2. Conditional Tabular GAN (CTGAN)

CTGAN [11] is a specialized form of Generative Adversarial Network (GAN) designed specifically for handling tabular data commonly found in databases and spreadsheets. Unlike traditional GANs that focus on generating graphics or text, CTGAN is tailored to create synthetic tabular data that closely emulates the statistical properties of the original data.

CTGAN trains both the generator and discriminator networks simultaneously to produce their respective outcomes. The generator network takes white noise as input and generates synthetic data samples. On the other hand, the discriminator network receives both real and synthetic data as input to differentiate between the two. As the training progresses, the generator network aims to produce synthetic data that increasingly resembles the actual data, while the discriminator network strives to improve its ability to distinguish between real and synthetic instances.

A key feature of CTGAN is its capability to generate conditioned synthetic data, enabling the generation of data under specific circumstances, such as predefined column values or the absence of certain patterns. This conditional data generation feature proves particularly valuable when adhering to regulatory or corporate guidelines during data synthesis.

CTGAN effectively generates synthetic tabular data while preserving the statistical characteristics of the original data and accommodating conditional data production. Consequently, it finds applications in various domains, including data privacy and security, data augmentation, and data sharing [12].

In this study, CTGAN was chosen over traditional GAN models [13] due to its ability to address the limitations of GAN in accurately capturing complex dependencies and distributions within structured data. By generating synthetic tabular data that closely resembles real-world data, CTGAN surpasses the scope of GAN primarily used for synthetic image generation. This makes CTGAN more suitable for intrusion detection in IoT networks [14].

## 3. Literature Review

Various studies have proposed different techniques for detecting DoS and DDoS attacks in IoT networks using machine learning algorithms. For instance, Cviti et al. developed a method using a boosting technique of logistic model trees to detect DDoS traffic for various classes of IoT devices. Their results achieved accuracy rates ranging from 99.92% to 99.9% for the four device classes considered [15]. Roopak et al. employed a convolutional neural network (CNN) with long short-term memory (LSTM) for classifying DDoS attacks and achieved high accuracy rates on the CISIDS-2017 datasets, with a precision of 99.26%, recall of 99.35%, and F1-score of 99.3% [16].

Hodo et al. proposed a multilayer perceptron (MLP)-based intrusion detection system (IDS) to detect DoS attacks in IoT networks, which accurately distinguished between various DDoS and DoS attacks [17]. Mohammed et al. proposed an IDS based on multiple ML algorithms, including decision tree (DT), k-nearest neighbors (k-NN), and Naive Bayes (NB), achieving accuracy rates of 100%, 98%, and 29%, respectively, using the CICIDS-2019 dataset samples [18].

Using the CIDDS-001, UNSWNB15, and NSL-KDD datasets, Verma et al. demonstrated a number of shallow ML algorithms, such as random forest (RF), Adaboost (AB), gradient boosting machine (GBM), extremely randomized trees (ERT), classification and regression tree (CART), and multilayer perceptron (MLP) neural network, with RF achieving the best results with an accuracy rate of 94% [19].

Chopra et al. compared several rudimentary ML algorithms, including Naive Bayes, J48, RF, and ZeroR classifiers, for detecting and classifying DDoS attacks in IoT using the Bot-IoT dataset. However, the authors suggest that these models may not perform well when applied to large-scale IoT datasets due to the poor accuracy performance of naive ML algorithms in such contexts [20].

In Churcher et al.’s work [21], the Bot-IoT dataset was used to conduct binary and multiclass classification tasks. They utilized weight-based class balancing techniques to produce balanced and asymmetrical representations of the data. The authors used Scikit-Learn [22] and Keras [23] with their default hyperparameters and reported on performance indicators such as precision and F1 score. The initial Bot-IoT dataset contained 35 variables, such as timestamps and the Argus sequence number; after removing columns with missing values, text, and unnecessary columns, the final dataset contained just 19. The percentage of the validation set used in the 80/20 data divide for training and testing was not disclosed. Using weighted datasets for binary classification in DDoS and DoS attack protocols, the ANN consistently outperformed other models with an accuracy of 99. When used for multiclass categorization, the ANN has the highest precision (97%) across all attack types in the Bot-IoT dataset.

Alimi et al. [24] introduced a revised RLSTM deep learning model to identify DoS attacks in IoT networks. They evaluated the proposed RLSTM model using two standard datasets: CICIDS-2017 and NSL-KDS. The experiments demonstrated that the proposed model substantially enhanced the detection accuracy, precision, recall, and F1 score.

Almaraz-Rivera et al. [25] conducted research on DoS attacks on IoT networks and created an intrusion detection system based on ML and deep learning models to analyze the Bot-IoT dataset. Using a variety of performance criteria, they found that the models were, on average, more accurate than 95% of the time, with the decision tree and MLP models being the best for detecting DDoS and DoS attacks in IoT networks.

Susilo and Sari (2020) [26] proposed the use of several machine-learning and deep-learning strategies, including random forests (RF), convolutional neural network (CNN), and multi-layer perceptron (MLP), for improving the security performance of IoT networks. The authors developed an algorithm for detecting denial-of-service (DoS) attacks using a deep-learning algorithm. The BoT-IoT dataset is used to evaluate their work, and they found that the deep-learning model could increase accuracy, making the mitigation of attacks that occur on an IoT network as effective as possible.

In their study, Kumar et al. introduced a fog computing-based distributed Intrusion Detection System (IDS) for detecting Distributed Denial of Service (DDoS) attacks on mining pools in IoT networks enabled by blockchain technology. The proposed model is evaluated using Random Forests and an optimized gradient tree boosting system on distributed fog nodes, and the evaluation is conducted using the BoT-IoT dataset. The results demonstrate that XGBoost performs better in binary attack detection, while the Random Forest outperforms in multi-attack detection. Furthermore, the Random Forest exhibits faster training and testing times on distributed fog nodes compared to XGBoost [27].

Table 2 shows the summary of related works

Table 2 presents an overview of different shallow ML algorithms used for detecting DDoS and DoS attacks in IoT devices. The findings indicate that DL algorithms, such as CNN and MLP, outperform shallow ML classifiers in terms of accuracy. However, it is worth noting that the choice of dataset plays a crucial role in determining the accuracy of the model.

For example, Verma et al. achieved the highest accuracy of 94% using random forest on the CIDDS-001, UNSW-NB15, and NSL-KDD datasets, while Mohammed et al. observed varying results when comparing naive Bayes, Bayes Net, and ZeroR on the UNSW-NB15 dataset. It’s essential to use datasets that reflect the characteristics of IoT networks, such as Bot-IoT and UNSW-NB15, to evaluate the effectiveness of existing and future approaches instead of using non-IoT datasets like CICIDS-2019, CISIDS-2017, and NSL-KDD.

Moreover, Table 2 highlights the lack of attention given to utilizing Generative Adversarial Networks (GAN) or its variants, such as CTGAN, for enhancing the detection of DDoS and DoS attacks on IoT networks.

## 4. Proposed Approach

**Figure 1:**
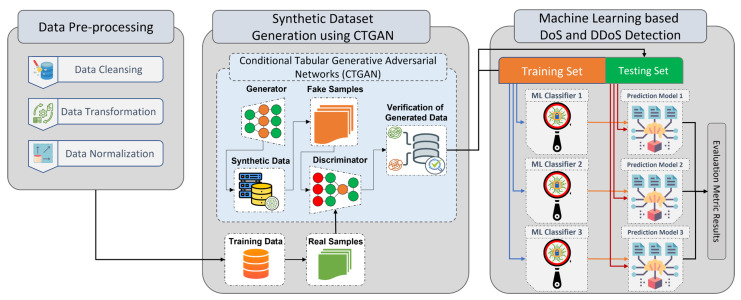
Proposed approach.

This section describes an approach to detect TCP and UDP DDoS and DoS attacks on IoT networks using CTGAN to produce adversarial samples that are highly representative of actual IoT network traffic. To further improve the accuracy of DDoS and DoS detection in IoT networks, these samples are used to train several shallow ML and DL classifiers. CTGAN can replicate data with near-perfect statistical accuracy since it is trained using real-world samples. Figure 1 depicts the three stages of the proposed approach: (1) data pre-processing, (2) synthetic data creation using CTGAN, and (3) machine learning-based DoS and DDoS detection. These three stages will be discussed in detail below.

### 4.1. Data Pre-Processing

Generally, data pre-processing helps improve the accuracy of models that use the data. In data pre-processing, it is essential to standardize or normalize the data to ensure that the features are on the same scale and have similar ranges. Without pre-processing, the accuracy of models that use the data can be compromised [28,29]. The measures taken to guarantee the quality of the dataset used in the research (refer to Section 5.1 for details about dataset used) are crucial. These measures include data cleaning, handling missing values, feature scaling, and transforming categorical variables. Failure to carry out these steps can lead to biased and unreliable results, rendering the entire research effort useless. Therefore, it is essential to prioritize data pre-processing to ensure accurate and reliable results. The explanation of measures carried out in this research is as follows:

- **Data cleansing:** This procedure involves identifying data that is lacking, incorrect, erroneous, or irrelevant so it can be updated or removed. For example, if a feature has no available value in the dataset, it is assigned a value of 0.
- **Categorical data transformation:** This step entails converting data from one format to another. For example, the characteristics of the String/Object datatype are substituted by a unique number. The Categorical data in the dataset used are: proto, saddr, sport, daddr, dport, category, subcategory. Table 3 shows sample of categorical data while Table 4 shows sample of categorical data transformation
- **Feature scaling:** This procedure maps the information onto the unit sphere or converts it to the interval [0, 1] (or any other interval). Table 5 show sample of feature scaling of used dataset. Using Equation (1), we max-min normalize the feature vector:
  (1) $$x_{i}=\frac{x_{i}-\min\left(x\right)}{\max(x)-\min(x)}$$

Data filtering is an important technique in data analysis that helps to extract meaningful information from large and complex datasets. By selecting a subset of the data that meets specific criteria or conditions, filtering can help to reduce noise and improve the accuracy of statistical and ML models.

Analysis of network traffic data is crucial for detecting TCP and UDP DDoS and DoS attacks in IoT networks. Filtering the dataset to include only TCP and UDP protocols is a vital step in this process since these are the most often utilized protocols in these attacks. The accuracy of the analysis and the dependability of the findings are both improved by filtering out unnecessary data, resulting in only a more focused and better-quality dataset.

### 4.2. CTGAN-Based Synthetic Data Generation

GAN shows impressive results in generating syntactic images which do not applicable to IDS. Therefore, several GAN variants such as Wasserstein GAN (WGAN) [30], TGAN [31], and CTGAN [11] are proposed to generate synthesizing tabular data that is suitable to evaluate the performance of IDS in detecting the presence of attacks In this research, CTGAN was chosen to generate a syntactic dataset as it demonstrated superior performance compared to WGAN and TGAN, as reported by Bourou et al. [32]. It has shown promise in various applications, including fraud detection, rare event detection, and anomaly detection.

This stage is the core stage of the proposed approach which aims to generate synthetic data and perturb it to create adversarial samples is a promising approach for improving the robustness of ML and DL learning models for detecting DoS and DDoS detection in IoT networks.

CTGAN generator network can be represented as a function G(X,Z), where *X* is the real data, and *Z* is a noise vector. The generator network inputs *X* and *Z* and produces a batch of synthetic data samples as outputs. The generator network is trained to minimize the distance between the distribution of the synthetic data and the real data distribution.

To create an adversarial example using CTGAN, a synthetic data sample $x_{syn}$ is chosen from the batch that is closest to the decision boundary between the current predicted class and the target class. A small perturbation is added to $x_{syn}$ to create an adversarial example $x_{adv}$. This perturbation can be represented as a function $P(x_{syn})$, where *P* is a function that adds a small amount of noise or changes to $x_{syn}$. The success of the adversarial example $x_{adv}$ is evaluated by computing the model’s output for $x_{adv}$ and comparing it to the target class *Y*. If the model misclassifies $x_{adv}$, it is considered a successful adversarial example. Additionally, to address the extreme class imbalance in the Bot-IoT dataset, this approach proposes using synthetic data generation as a solution. The output of this stage is $x_{adv}$, which is used as input for the next stage.

The workflow of the proposed approach can be summarized as follows:

Synthetic Traffic Generation: The CTGAN-based IDS employs a generator network to produce synthetic traffic that closely mimics legitimate traffic patterns. This synthetic traffic generation step enables the IDS to effectively distinguish between legitimate and malicious traffic, facilitating accurate detection and mitigation of DoS and DDoS attacks.

Discriminator Network: The discriminator network, a crucial component within the CTGAN framework, learns to differentiate between legitimate and malicious traffic. By analyzing the characteristics and patterns of the traffic, the discriminator enhances the IDS’s ability to detect and classify attacks. This helps in effectively identifying and mitigating both DoS and DDoS attacks on IoT networks.

Enhanced Detection Models: The syntactic tabular data generated by CTGAN is utilized to train multiple shallow machine-learning and deep-learning classifiers. The training process involves using the synthetic data to enhance the performance of the detection models. This results in improved accuracy and effectiveness in detecting and mitigating DoS and DDoS attacks.

The proposed approach leverages the capabilities of CTGAN to generate synthetic traffic, train detection models, and enhance the overall performance of the IDS. By combining synthetic traffic generation, discrimination analysis, and improved detection models, the approach aims to enhance the security of IoT networks against DoS and DDoS attacks.

### 4.3. DoS and DDoS Attack Detection

During this phase, the focus is on training multiple shallow ML and DL models to create detection models capable of accurately detecting DDoS and DoS attacks. The adversarial examples generated in the previous stage, denoted as $x_{adv}$, are utilized as training data for these models. The main output of this phase is the resulting trained models, which can be deployed online to detect DDoS and DoS attacks in IoT networks.

It is worth noting that this approach is not limited to TCP and UDP DDoS and DoS attacks. It can be applied to various datasets, enabling the identification of different types of attacks across different domains or fields. The flexibility of this approach makes it adaptable and applicable to diverse scenarios where attack detection is required.

By training multiple models using the adversarial examples, the aim is to enhance the detection capabilities and robustness of the models against various attack scenarios. Once deployed, these trained models can effectively analyze network traffic data and accurately identify instances of DDoS and DoS attacks, contributing to the security and stability of IoT networks. The workflow of the proposed approach in detecting DDoS/DOS attacks in IoT network can be summarized as follows:

- The CTGAN-based IDS employs a generator network to produce synthetic traffic that closely mimics legitimate traffic patterns. This synthetic traffic generation step enables the IDS to effectively distinguish between legitimate and malicious traffic, facilitating accurate detection and mitigation of DoS and DDoS attacks.
- The discriminator network, a crucial component within the CTGAN framework, learns to differentiate between legitimate and malicious traffic. By analyzing the characteristics and patterns of the traffic, the discriminator enhances the IDS’s ability to detect and classify attacks. This helps in effectively identifying and mitigating both DoS and DDoS attacks on IoT networks.
- The syntactic tabular data generated by CTGAN is utilized to train multiple shallow machine-learning and deep-learning classifiers. The training process involves using the synthetic data to enhance the performance of the detection models. This results in improved accuracy and effectiveness in detecting and mitigating DoS and DDoS attacks.

## 5. Experimental Results

This section describes the experimental setup, the data, the evaluation metrics, and the results of the proposed approach.

### 5.1. Dataset

The BoT-IoT dataset [33] is employed to assess the proposed method’s capability in detecting TCP and UDP DDoS and DoS attacks. This dataset, created by the Cyber Range Lab at The Center of UNSW Canberra Cyber, emulates a realistic network environment and encompasses both regular and botnet traffic in formats such as PCAP, argus, and CSV files. The complete dataset comprises over seventy-two million records, while a 10% subset contains approximately three million records. For our experiments, we utilized a 5% subset of the dataset, focusing on the top ten features. The BoT-IoT dataset was chosen due to its widespread use in existing research such as in [27,34]. It is a commonly utilized dataset that provides a comprehensive representation of various IoT network traffic scenarios. Researchers frequently rely on the BoT-IoT dataset for benchmarking intrusion detection systems and evaluating the performance of detection algorithms.

The number of records in the training and testing sets for each attack category in the BoT-IoT traffic is presented in Table 6. These attacks are classified into seven main categories, which are further mapped into five categories, as illustrated in Table 7. The information matrix of the training and testing datasets is depicted in Table 8 and Table 9, respectively.

**Figure 2:**
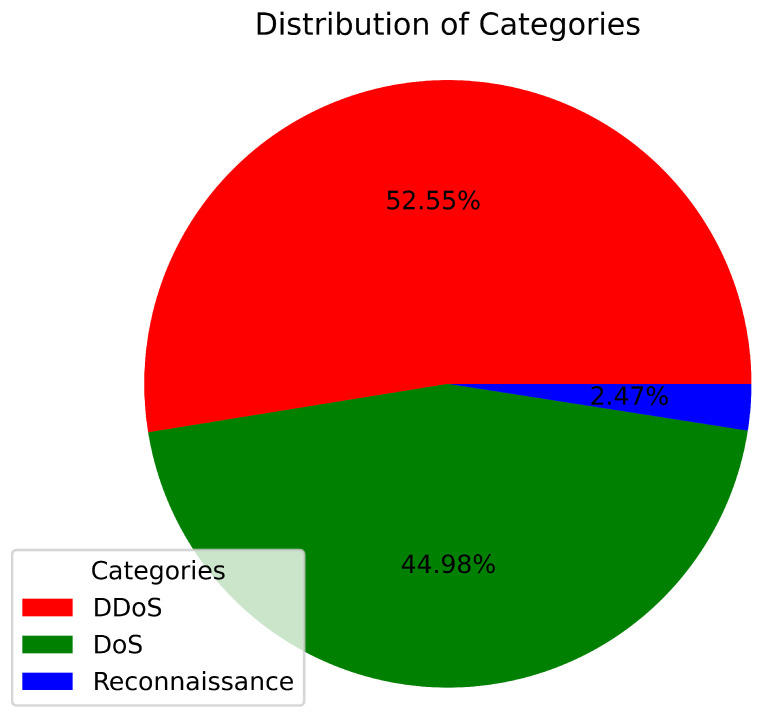
Proportion of DDoS and DoS attacks in the BoT-IoT dataset.

The analysis of Table 6 reveals that UDP and TCP attacks are the predominant attack types within the 5% subset of the BoT-IoT dataset. Furthermore, Table 7 highlights that DDoS and DoS attacks constitute the majority of attacks in the BoT-IoT dataset. Hence, this research focuses on detecting UDP and TCP DDoS and DoS attacks in IoT networks. The distribution of DDoS and DoS attacks in the BoT-IoT dataset is visualized in Figure 2. Additionally, Table 10 presents the attack category distribution of the BoT-IoT dataset after applying the filtering process.

The pre-processed filtered dataset presented in Table 10 serves as the input for CTGAN to generate the syntactic dataset $x_{adv}$. In order to enable binary classification for shallow ML and DL classifiers, the DDoS and DoS categories are merged into a single category, labeled as 1, while the “normal” category is retained and labeled as 0. Consequently, the generated dataset consists of two primary classes: Attack (1) and normal (0). The distribution of attack categories in the synthetic dataset is shown in Table 11.

It is worth mentioning that during the transformation steps, the categorical data is converted to numerical values, as shown in Table 11. Moreover, the number of normal instances has increased from 118 to 441,101 instances. This increase in the number of normal instances solves the problem of severe imbalanced data in the BoT-IoT dataset. The generated dataset is used to train several shallow ML and DL classifiers.

### 5.2. Evaluation Metrics

Described below are metrics for measuring the efficacy of the proposed approach. Table 12 displays the evaluation metrics based on the various properties of the confusion matrix.

Numerous research studies, including [35,36], use the metrics employed here to evaluate the efficacy of IDS. The evaluation of the proposed approach requires the computation of all of these measures.

### 5.3. Results and Discussion

The objective of this section is to evaluate the efficiency of the syntactic tabular dataset, denoted as $x_{adv}$, which was generated using CTGAN, in improving the performance of detection models. To achieve this, we trained several shallow ML classifiers, namely Logistic Regression (LR) [37], Naive Bayes (NB) [38], Random Forest (RF) [39], Decision Tree (DT) [40], and Support Vector Machine (SVM) [41]. Additionally, we trained several deep learning classifiers, namely Long Short-Term Memory (LSTM) [42], Recurrent Neural Network (RNN) [43], and Gated Recurrent Units (GRUs) [44]. These classifiers were trained using the $x_{adv}$ dataset and evaluated using an unseen testing dataset (5% testing dataset). The default parameters were used for shallow ML classifiers, while the parameters for DL classifiers were based on [45].

Table 13 presents the evaluation results of these models using the BoT-IoT dataset, while Table 14 presents the evaluation results using the synthetic dataset generated by CTGAN. These evaluation metrics provide insights into the effectiveness of the $x_{adv}$ dataset in enhancing the performance of the detection models.

Table 13 presents the performance metrics of different models. The logistic regression, Naive Bayes, and SVM models have similar performance metrics, including a detection accuracy of 0.699, precision ranging from 0.367 to 0.849, recall score of 0.699, and F1 measure of 0.823. Similarly, the random forest classifier and decision tree classifier models share the same performance metrics, with a detection accuracy of 0.648, precision of 0.342, recall score of 0.683, and F1 measure of 0.786. Among all the models, the LSTM model demonstrates superior performance with a detection accuracy of 0.978, precision of 0.966, recall score of 1.0, and F1 measure of 0.984. On the other hand, the RNN and GRU models exhibit lower performance metrics, with a detection accuracy of 0.693 and 0.695, respectively, precision of 0.356 and 0.359, recall score of 0.698, and F1 measure of 0.819 and 0.820, respectively.

Table 14 displays the performance metrics of different models. The LSTM, RNN, and GRU models demonstrate the highest performance metrics in terms of detection accuracy, precision, recall score, and F1 measure. Specifically, the LSTM model achieves the highest detection accuracy of 0.994 and F1 measure of 0.996. The RNN and GRU models also exhibit strong performance, with a detection accuracy of 0.986, precision of 0.978, recall score of 1.000, and F1 measure of 0.990 and 0.986, respectively. The Naive Bayes and SVM models perform moderately well, with F1 measure scores of 0.9754 and 0.8086, respectively. On the other hand, the random forest classifier and decision tree classifier models show lower detection accuracy and F1 measure scores, indicating their limited effectiveness in detecting DoS and DDoS attacks in IoT networks. Table 15 highlights the enhancements achieved by CTGAN for each of the shallow ML and DL classifiers, emphasizing the improvements in the detection models when utilizing the syntactic tabular dataset generated by CTGAN.

Based on Table 15, it is observed that the models exhibit varying degrees of enhancement in their performance compared to the results listed in Table 13. For shallow ML classifiers, the NB model shows the highest enhancement in detection accuracy with a score of 0.267, followed by LR with a score of 0.193, and DT with a score of 0.183. SVM, on the other hand, has the lowest enhancement in detection accuracy with a score of 0.076. Regarding DL classifiers, the RNN model shows the highest enhancement in detection accuracy with a score of 0.293, followed by GRU with the same score. LSTM, however, has the lowest enhancement in detection accuracy with a score of 0.016. The table also presents the enhancement in precision, recall score, and F1 measure. It is observed that the NB model shows the highest enhancement in precision with a score of 0.598. RNN and GRU models exhibit the highest enhancement in recall score with a score of 0.302, while the LSTM model shows a negligible enhancement in recall score with a score of −0.001. The NB model also shows the highest enhancement in the F1 measure with a score of 0.1524, while RF exhibits a negligible enhancement with a score of −0.0095.

In summary, the results indicate that the CTGAN approach has a positive impact on the performance of both shallow ML and DL classifiers in most cases. Specifically, the NB, RNN, and GRU models demonstrate notable improvements in detection accuracy, precision, recall, and F1 measure when using the syntactic tabular dataset generated by CTGAN.

### 5.4. Discussion

Overall, the findings of this study reveal the significant impact of leveraging CTGAN (Conditional Table GAN) on the performance of intrusion detection models in IoT networks. By using CTGAN to generate synthetic attack instances and augment the training dataset, the LSTM, RNN, and GRU models have exhibited remarkable improvements in accurately detecting DoS and DDoS attacks. This highlights the importance of utilizing advanced data generation techniques to enhance the effectiveness of intrusion detection systems.

In contrast, the random forest classifier and decision tree classifier models have demonstrated comparatively weaker performance metrics in this study. These models, when trained on the original dataset without the benefits of CTGAN-generated synthetic data, may lack the ability to effectively capture the intricacies and complexities of modern IoT-based attacks. Therefore, caution is advised when considering the use of these models for intrusion detection in IoT networks.

To ensure robust and reliable intrusion detection, it is recommended to prioritize the utilization of LSTM, RNN, and GRU models, which have shown superior performance in accurately identifying DoS and DDoS attacks. These models, when combined with advanced data generation techniques like CTGAN, have the potential to significantly enhance the security of IoT networks against evolving attack vectors. Furthermore, a comparative analysis was conducted between our proposed approach and the work proposed in [26]. In order to ensure a fair comparison, the CNN and MLP models were implemented using the parameter settings specified in [26]. Subsequently, the performance of the CNN and MLP models was evaluated using the same batch sizes and epoch values as mentioned in [26].

Table 16 compares the results of the CNN and MLP models reported in [26] with the results obtained using CTGAN-generated synthetic data (batch size 32, varied epoch sizes). Similarly, Table 17 shows the comparison for batch size 64, and Table 18 for batch size 128.

The results tabulated in Table 16, Table 17 and Table 18 indicate that the proposed models consistently outperform the models reported in [26] in terms of mean accuracy in the majority of cases. This strongly suggests that the proposed approach, which utilizes CTGAN-generated synthetic data, significantly improves the detection accuracy of both CNN and MLP models across various batch sizes and epochs.

These findings highlight the effectiveness of the proposed approach in enhancing the performance of intrusion detection models. By leveraging CTGAN to generate synthetic data, the CNN and MLP models achieve higher mean accuracy, indicating their improved capability to accurately detect intrusion attempts in IoT networks. This improvement can be attributed to the ability of CTGAN to generate realistic synthetic data that captures the complexities of modern attack patterns, enabling the models to learn more effectively and make better predictions.

Overall, these results provide strong evidence supporting the efficacy of the proposed approach in improving the performance of intrusion detection systems. The use of CTGAN-generated synthetic data offers a promising avenue for enhancing the accuracy and reliability of detection models, ultimately contributing to the security and resilience of IoT networks against evolving cyber threats.

## 6. Conclusions and Future Works

Our study proposes a CGAN-based IDS for detecting DDoS attacks on IoT networks. The proposed IDS overcomes the limitations of existing IDS systems by employing a generator network to create synthetic traffic that imitates legitimate traffic patterns, and a discriminator network to detect anomalies. We evaluated the proposed approach using the BoT-IoT dataset in two scenarios.

In the first scenario, we evaluated multiple machines and deep learning classifiers using the original BoT-IoT dataset. This allowed us to establish a baseline performance for the detection models. In the second scenario, we evaluated the same machines and classifiers using the syntactic tabular dataset generated by CTGAN. This enabled us to assess the impact of using synthetic data on the performance of the detection models.

Our experimental results indicate that the syntactic tabular dataset significantly enhanced the detection model performance of multiple machines and deep learning classifiers. The use of synthetic data generated by CTGAN improved the models’ ability to accurately detect DDoS attacks on IoT networks. These findings demonstrate the effectiveness of our proposed CGAN-based IDS in improving the performance of intrusion detection systems.

In future work, we plan to investigate the effectiveness of our proposed CGAN-based IDS for detecting other types of attacks on IoT networks. By expanding the scope of our research, we aim to develop a comprehensive IDS solution that can effectively detect various types of intrusions in IoT environments. Additionally, we also aim to explore the potential of utilizing reinforcement learning techniques to further enhance the performance of the proposed Intrusion Detection System (IDS). By leveraging the capabilities of reinforcement learning, we anticipate achieving even higher accuracy and adaptability in detecting and mitigating attacks targeting IoT devices. Furthermore, we plan to evaluate the performance of the proposed approach using various benchmarking datasets to ensure its effectiveness and robustness across different scenarios.

## Figures and Tables

**Table 1:** Attacks on IoT networks other than DDoS and DoS.

Attack Type	Explanation
Malware and Ransomware	Malicious programs that are downloaded and installed on IoT gadgets and then cause damage, steal information, or turn the gadgets into part of a botnet. Data on a device is encrypted and then locked until a ransom is paid
Man-in-the-Middle (MitM)	The connection between IoT devices and the network may be intercepted by hackers, allowing them to eavesdrop, alter data, or insert harmful instructions. This leads to compromised security, altered data, or outright device control.
Physical Attacks	Physically accessing or tampering with an IoT device with the intent of stealing data, changing its behavior, or obtaining control over it. To identify and prevent such attacks, strong physical security measures are required.
Privilege Escalation	Gaining administrative access by exploiting flaws in the software or configuration of an IoT device. Because of this, malicious actors may get access to private information, change the way a device normally operates, or even go beyond its limits.
Information Leakage	The disclosure of private information, such as user passwords, configuration settings, or personal data, by IoT devices without permission. Those who would steal identities or get access illegally or maliciously take advantage of this vulnerability.
Replay Attacks	A method of recording and then playing back authorized interaction between IoT gadgets. Because to this, malicious acts, entry into protected regions, and authentication bypass are all possible.
DNS Attacks	DNS hijacking is the practice of diverting traffic from legitimate websites to malicious ones. Because of this, unauthorized parties may gain access to or modify information sent from an IoT device to its intended recipient.
Firmware Attacks	Taking advantage of security holes in the firmware of embedded systems used in IoT devices. Software that has been compromised may be used to take over a device, modify its behavior, or install malicious software. The security and functioning of a device may be severely compromised by an attack on its firmware.

**Table 2:** Summary of related works.

Reference	Algorithm	Dataset	Accuracy
[15]	logistic model trees	IoT device classes	99.92% to 99.99%
[16]	Convolutional neural network (CNN) with LSTM	CISIDS-2017	99.03%
[17]	Multi-layer perceptron (MLP)	Various types of DDoS and DoS attacks	High accuracy
[18]	DT, k-NN, and NB	CICIDS-2019	100%, 98%, 29%
[19]	RF, AB, GBM, ERT, CART, and MLP	CIDDS-001, UNSWNB15, NSL-KDD	94% (RF)
[20]	Naive Bayes, Bayes Net, ZeroR	UNSW-NB15	Varying results
[21]	Artificial Neural Networks (ANN)	BoT-IoT	99% (binary class) and 97% (multiclass class)
[24]	Refined long short-term memory (RLSTM) deep learning model	CICIDS-2017 and NSL-KDS	Outperforms other methods
[25]	Machine Learning and Deep Learning models (Decision Tree and Multi-layer Perceptron)	Bot-IoT	Average accuracy over 99%
[26]	CNN Multi-layer Perceptron RF	Bot-IoT	Average accuracy 92.85%
[27]	Random Forest XGbooest	Bot-IoT	Average accuracy 99%

**Table 3:** Sample of categorical data.

Proto	Saddr	Sport	Daddr	Dport	Category	Subcategory
udp	192.168.100.150	6551	192.168.100.3	80	DDoS	UDP
tcp	192.168.100.150	5532	192.168.100.3	80	DDoS	TCP
tcp	192.168.100.147	27,165	192.168.100.3	80	DDoS	TCP
udp	192.168.100.150	48,719	192.168.100.3	80	DoS	UDP
udp	192.168.100.147	22,461	192.168.100.3	80	DDoS	UDP

**Table 4:** Sample of categorical data transformation.

Proto	Saddr	Sport	Daddr	Dport	Category	Subcategory
4	4	61,685	13	4191	0	7
3	4	50,363	13	4191	0	6
3	1	19,080	13	4191	0	6
4	4	43,028	13	4191	1	7
4	1	13,854	13	4191	0	7

**Table 5:** Sample of feature scaling of used dataset.

pkSeqID	Proto	Saddr	Sport	Daddr	Dport	Seq
0.856684	1.00	0.266667	0.941181	0.265306	0.887924	0.961012
0.663009	0.75	0.266667	0.768431	0.265306	0.887924	0.979089
0.538722	0.75	0.066667	0.291120	0.265306	0.887924	0.239964
0.338217	1.00	0.266667	0.656515	0.265306	0.887924	0.378203
0.888094	1.00	0.066667	0.211382	0.265306	0.887924	0.400685

**Table 6:** Attack distribution in the training and testing datasets in BoT-IoT traffic.

Attack Type	Training Dataset	Testing Dataset
UDP	566,132	396,580
TCP	455,737	318,337
Service_Scan	20,788	14,542
OS_Fingerprint	5058	3621
HTTP	721	504
Normal	118	107
Keylogging	20	14
Data_Exfiltration	1	0
Total	1,048,575	733,705

**Table 7:** Attack Category Distribution in BoT-IoT Traffic.

Category	Training Dataset	Testing Dataset
DDoS	550,955	385,309
DoS	471,635	330,112
Reconnaissance	25,846	18,163
Normal	118	107
Theft	21	14
Total	1,043,575	733,705

**Table 8:** Information matrix of training dataset.

Column Name	Count	Mean	Std	Min	Max
pkSeqID	1,048,575	1,833,736	1,058,796	5.0	3,668,519
seq	1,048,575	121,283.3	75,795.08	1.0	262,207
stddev	1,048,575	0.886813	0.803454	0.0	2.496763
N_IN_Conn _P_SrcIP	1,048,575	82.58135	24.36642	1.0	100.0
min	1,048,575	1.019018	1.484272	0.0	4.980471
state_number	1,048,575	3.134601	1.186406	1.0	11.0
mean	1,048,575	2.231664	1.517782	0.0	4.981882
N_IN_Conn _P_DstIP	1,048,575	92.48208	18.13428	1.0	100.0
drate	1,048,575	0.457156	67.19496	0.0	58,823.53
srate	1,048,575	3.497612	1058.112	0.0	1,000,000.0
max	1,048,575	3.020940	1.860618	0.0	4.999999
attack	1048575.0	0.9998875	0.0106076	0.0	1.0

**Table 9:** Information matrix of testing dataset.

Column Name	Count	Mean	Std	Min	Max
pkSeqID	733,705	1,834,472	1,058,826	2.0	3,668,507
seq	733,705	121,412.819892	75,823.39884	1.0	262,212
stddev	733,705	0.887894	0.804013	0.0	2.496758
N_IN_Conn _P_SrcIP	733,705	82.492551	24.426145	1.0	100.0
min	733,705	1.018868	1.484235	0.0	4.980470
state_number	733,705	3.135073	1.186427	1.0	11.0
mean	733,705	2.233429	1.517572	0.0	4.981785
N_IN_Conn _P_DstIP	733,705	92.427763	18.216076	1.0	100.0
drate	733,705	0.506298	74.330175	0.0	58,823.53
srate	733,705	2.262398	403.408092	0.0	333,333.3125
max	733,705	3.023000	1.860725	0.0	4.999999
attack	733,705	0.999854	0.012075	0.0	1

**Table 10:** Attack Category Distribution of BoT-IoT Dataset After Applying the Filtering.

Category	Protocol	Number of Records
DDoS	TCP	279,601
	UDP	271,056
Total of DDoS records		550,657
DoS	TCP	295,063
	UDP	176,123
Total of DoS records		471,186
Normal	TCP	92
	UDP	13
	ARP	10
	IPV6-ICMP	3
Total of Normal records		118
Total of records		1,021,961

**Table 11:** Attack category distribution of the synthetic dataset.

Category	Traffic Type	Number of Packets
0 (normal)	4 (TCP)	347,715
	3 (UDP)	94,386
1 (attack)	4 (TCP)	313,836
	3 (UDP)	244,063
Total number of records		1,000,000

**Table 12:** Evaluation metrics.

Evaluation Metric	Definition
True positive (TP)	Conditions under which the classifier makes the right decision an attack
False negative (FN)	This is a condition in which the classifier incorrectly labels an attack as normal.
False positive (FP)	Refers to situations in which the classifier incorrectly identifies a normal instance as an attack.
True negative (TN)	This is the situations in which the classifier makes the right call common occurrences
Precision	The ratio of accurately predicted attacks to all samples predicted as attacks. Precision = TP / (TP + FP)
Recall / Detection Rate	The proportion of all attack samples correctly classified as attacks vs. all attack samples. Recall = TP / (TP + FN)
False Alarm Rate / False Positive Rate	The ratio of incorrectly predicted attack samples vs. all normal samples. False Alarm Rate = FP / (TN + FP)
True Negative Rate	The proportion of correctly classified normal samples vs. all normal samples. True Negative Rate = TN / (TN + FP)
Accuracy	The proportion of instances correctly classified vs. the total number of instances. Accuracy = (TP + TN) / (TP + TN + FP + FN)
F1-measure	The harmonic means of precision and recall. F1 Measure = 2 × (Precision x Recall) / (Precision + Recall)

**Table 13:** Evaluation results using the BoT-IoT dataset.

Model	Detection Accuracy	Precision	Recall Score	F1 Measure
LR	0.699	0.367	0.699	0.823
NB	0.699	0.351	0.699	0.823
RF	0.648	0.342	0.683	0.786
DT	0.648	0.342	0.683	0.786
SVM	0.699	0.849	0.699	0.823
LSTM	0.978	0.966	1.000	0.984
RNN	0.693	0.356	0.698	0.819
GRU	0.695	0.359	0.698	0.820

**Table 14:** Evaluation results using a synthetic dataset generated by CTGAN.

Model	Detection Accuracy	Precision	Recall Score	F1 Measure
LR	0.892	0.868	1.0	0.9170
NB	0.966	0.949	1.0	0.9754
RF	0.744	0.770	1.0	0.7765
DT	0.831	0.820	1.0	0.8629
SVM	0.775	0.786	1.0	0.8086
LSTM	0.994	0.991	0.999	0.996
RNN	0.986	0.978	1.0	0.990
GRU	0.981	0.971	1.0	0.986

**Table 15:** Enhancements made by CTGAN for each shallow ML and DL classifiers.

Model	Detection Accuracy	Precision	Recall Score	F1 Measure
LR	0.193	0.501	0.301	0.094
NB	0.267	0.598	0.301	0.1524
RF	0.096	0.428	0.317	−0.0095
DT	0.183	0.478	0.317	0.0769
SVM	0.076	−0.063	0.301	−0.0144
LSTM	0.016	0.025	−0.001	0.012
RNN	0.293	0.622	0.302	0.171
GRU	0.286	0.612	0.302	0.166

**Table 16:** The result of batch size 32.

Epoch	Work in [26]	Mean Accuracy	Proposed Work	Mean Accuracy
10	CNN	90.85%	CNN	97.48%
10	MLP	53.07%	MLP	97.63%
30	CNN	89.82%	CNN	83.65%
30	MLP	62.95%	MLP	97.37%
50	CNN	88.30%	CNN	79.09%
50	MLP	62.00%	MLP	97.23%

**Table 17:** The result of batch size 64.

Epoch	Work in [26]	Mean Accuracy	Proposed Work	Mean Accuracy
10	CNN	91.15%	CNN	96.86%
10	MLP	76.92%	MLP	97.25%
30	CNN	91.02%	CNN	80.20%
30	MLP	54.04%	MLP	97.49%
50	CNN	90.64%	CNN	80.11%
50	MLP	53.89%	MLP	97.28%

**Table 18:** The result of batch size 128.

Epoch	Work in [26]	Mean Accuracy	Proposed Work	Mean Accuracy
10	CNN	90.87%	CNN	95.17%
10	MLP	54.10%	MLP	97.20%
30	CNN	90.76%	CNN	79.97%
30	MLP	54.43%	MLP	97.16%
50	CNN	91.27%	CNN	80.96%
50	MLP	79.01%	MLP	97.18%

## Data Availability

Not applicable.
